# Towards community-based and recovery-oriented care for severe mental disorders in Southern and Eastern Europe: aims and design of a multi-country implementation and evaluation study (RECOVER-E)

**DOI:** 10.1186/s13033-020-00361-y

**Published:** 2020-04-22

**Authors:** Laura Shields-Zeeman, Ionela Petrea, Filip Smit, Bethany Hipple Walters, Jovo Dedovic, Martina Rojnic Kuzman, Vladimir Nakov, Raluca Nica, Antoni Novotni, Catharina Roth, Aleksandar Tomcuk, Ben F. M. Wijnen, Michel Wensing

**Affiliations:** 1 Trimbos Institute (Netherlands Institute of Mental Health and Addiction), P.O. Box 725, 3500 AS Utrecht, The Netherlands; 2 Centre for Economic Evaluation, Trimbos Institute (Netherlands Institute of Mental Health and Addiction), Utrecht, The Netherlands; 3 Department of Public Mental Health and Prevention, Trimbos Institute (Netherlands Institute of Mental Health and Addiction), Utrecht, The Netherlands; 4 Department of Biostatistics and Epidemiology and Department of Clinical, Neuro and Developmental Psychology, Amsterdam Public Health Research Institute, VU University Medical Center, Amsterdam, The Netherlands; 5 Special Psychiatric Hospital Dobrota, Kotor, Montenegro; 6 Zagreb University Hospital Centre, Zagreb, Croatia; 7 National Center for Public Health Analyses, Sofia, Bulgaria; 8 Romanian League for Mental Health, Bucharest, Romania; 9 University Clinic of Psychiatry, Skopje, North Macedonia; 10 Department of General Practice and Health Service Research, University Hospital Heidelberg, Heidelberg, Germany

**Keywords:** Community mental health, Recovery, Severe mental illness, Implementation research, Public mental health, Eastern Europe

## Abstract

**Background:**

Substantial strides have been made around the world in reforming mental health systems by shifting away from institutional care towards community-based services. Despite an extensive evidence base on what constitutes effective care for people with severe mental ill-health, many people in Europe do not have access to optimal mental health care. In an effort to consolidate previous efforts to improve community mental health care and support the complex transition from hospital-based to community-based care delivery, the RECOVER-E (LaRge-scalE implementation of COmmunity based mental health care for people with seVere and Enduring mental ill health in EuRopE) project aims to implement and evaluate multidisciplinary community mental health teams in five countries in Central and Eastern Europe. This paper provides a brief overview of the RECOVER-E project and its methods.

**Methods:**

Five implementation sites were selected (Sofia, Bulgaria; Zagreb, Croatia; Skopje, North Macedonia; Kotor, Montenegro; Siret-Suceava, Romania) where hospital-based mental health services are available (care as usual, CAU) for patients with severe mental disorders (severe depression, bipolar disorder, schizophrenia). The intervention consists of the introduction of a new service delivery model in each site, consisting of community-based recovery-oriented care delivered by trained multidisciplinary community mental health teams (including a peer worker with lived experience of a severe mental disorder). The implementation outcomes of the teams and the effect of the team’s approach on patient and service utilisation outcomes will be evaluated using a mix of research methods. The study includes five planned hybrid implementation-effectiveness trials (1 per site) with patient-level randomization (n = 180, with patients randomised to either care as usual or intervention condition). Effectiveness is evaluated using a pragmatic non-blinded design with patients randomised into two parallel groups: receiving new community-based care or receiving usual care in the form of institutional, hospital-based mental health care. Trial-based health economic evaluation will be conducted; implementation outcomes will be evaluated, with data aligned with dimensions from the RE-AIM framework. Pathways to sustaining project results will be developed through policy dialogue sessions, which will be carried out in each country and through ongoing policy engagement activities at the European level.

**Discussion:**

The RECOVER-E project has been developed and conducted to demonstrate the impact of implementing an evidence-based service delivery model for people with severe mental illness in different contexts in middle-income countries in Central and Eastern Europe. It is expected that the results will contribute to the growing evidence-base on the health and economic benefits of recovery-oriented and community-based service models for health systems in transition.

*Trial registration* Each trial was registered before participant enrolment in the clinicaltrials.gov database: Site—Croatia, Zagreb (Trial Reg. No. NCT03862209); Montenegro, Kotor (Trial Reg. No. NCT03837340); Romania, Suceava (Trial Reg. No. NCT03884933); Macedonia, Skopje (Trial Reg. No. NCT03892473); Bulgaria, Sofia (Trial Reg. No. NCT03922425)

## Background

In order to improve the quality of care and outcomes of those with a mental health problem, many mental health systems may need reform. Extensive developments have been made around the world in reforming mental health systems, particularly through policy and services development, by shifting away from institutional care towards community-based services [1–3]. However, these strides are often not sufficient to result in marked changes in mental health care. These challenges particularly affect vulnerable populations such as people with severe mental health problems, who may require a complex mix of services, and may be considered as high-cost, high need populations in usage of health and social care services. Despite an extensive evidence base on what constitutes effective care for people with severe mental ill health, many people living in Europe do not have access to optimal mental health care [4, 5]. As mental disorders continue to pose preventable disability among a substantial number of people in Europe [6, 7], access to care and support is essential in ensuring people with mental health problems have the potential to lead fulfilling lives [8]. Mental health services inclusive of medical, psychological and social support have been shown to improve health-related outcomes (e.g. treatment adherence, remission, quality of life, personal and social functioning) as well as social outcomes (e.g. reduced social stigma, increased housing stability, vocational rehabiltiation and community participation) [4, 9–11].

Research has shown that people with severe mental ill health prefer services provided in their own environments in order to maintain relationships and employment. As a result, personal and social recovery is facilitated when support is provided within a comprehensive network in and around a person’s community [12, 13]. Service users have reported fewer negative symptoms, enhanced social capital, and greater life satisfaction when the process of deinstitutionalisation is conducted through a strong network of community-based services, to support the transition from the hospital to the community [12–15].

Healthcare professionals and policymakers who implement mental health system reforms often focus on revising policy and legislative frameworks for mental health, deploying training or quality improvement programs for specialist and non-specialised health professionals, and piloting new delivery models [1, 3, 16–20]. Due to limited resources, including financial and professional capacity limitations, large-scale implementation and evaluation of community-based mental health services remains a challenge for many national health care systems; in particular, there are few studies of service delivery changes in mental health care in Central and Eastern Europe [21]. This absence of evidence-based care pathways and referral systems may suggest that community-based mental health services have not been prioritized in many Central and Eastern European countries, as care remains primarily provided within institutions [22]. Community-based care is not commonly available or operational, in many low and middle income countries [23, 24], including in the countries involved in the RECOVER-E project.

In an effort to consolidate previous efforts to improve community mental health care and support the complex transition from hospital-based to community-based care provision, the RECOVER-E (La**R**ge-scal**E** implementation of **CO**mmunity based mental health care for people with se**V**ere and **E**nduring mental ill health in Eu**R**op**E**) project aims to implement well-functioning multidisciplinary community mental health teams in five sites in five countries in Central and Eastern Europe. These care teams will serve as the central node for the coordination and provision of care for people with severe mental illness (SMI) at one care location in each country. Care coordination and referral pathways will be detailed in treatment protocols and workflows that will serve to guide future services expansion in the country and/or be used in policy and plans by local, regional, and national decision-makers.

The overall goal of the RECOVER-E project is to contribute to the implementation of and research on an evidence-based community-based service delivery model for recovery-oriented care in five sites in middle-income countries (Croatia, Montenegro, North Macedonia, Bulgaria, and Romania) to improve functioning, quality of life, and mental health outcomes for people with severe and enduring mental ill health (such as schizophrenia, bipolar disorder, and/or severe depression).

The specific sub-goals of the RECOVER-E project are:

- designing, implementing, and evaluating recovery-oriented care for people with severe mental illness in community settings;
- recognizing the value of experiential knowledge through including peer experts as members of community mental health teams;
- identifying intervention and program elements, as well as contextual factors, which enhance sustainable implementation of community-based mental healthcare for people with severe mental illness; and
- developing scale-up plans for regional and national decision-makers, as informed by the intervention’s implementation and impact, for sustained implementation and scale up after the research study’s funded timeline.

The purpose of this manuscript is to present the protocol for the RECOVER-E project’s research study. The specific research objectives of the RECOVER-E project are:

- testing the impact of the intervention on patient outcomes and service utilisation outcomes;
- gathering in-depth data on implementation processes, which includes identifying key contextual factors, barriers, and facilitators of implementation; and
- assessing the cost-effectiveness of the intervention compared to usual care.

We hypothesise that the introduction of community-based service delivery models for providing care to people with severe mental illness will contribute to an improvement of functioning and quality of life for people with severe mental illness, as well as to fewer admissions and re-admissions to inpatient psychiatric care. As further described in “Methods” section of this manuscript, any improvements of functioning and quality of life will be measured using previously tested, internationally recognized scales, surveys, and instruments. Data on admissions and re-admissions will be collected from hospital and institutional records at each site, using standardized methods of data capture.

The outcomes and results of the research on the community-based service delivery in the five sites will be shared with policymakers, stakeholders in Central and Eastern Europe, healthcare providers, and researchers through policy dialogues, conference presentations, briefings, reports, and journal articles.

## Methods

### Study design

In order to understand the processes of the teams and the impact on care, the work of the teams will be evaluated using a mix of research methods. First, five hybrid effectiveness-implementation trials have been devised (1 per trial site) to assess the effects of the teams and of the delivery of new team-based community mental health care approach on implementation outcomes (i.e. the coverage and fidelity of evidence-based care at the health system level) and on patient-level outcomes (i.e. health gains in terms of improved role functioning and better health-related quality of life). These five trials have a pragmatic, non-blinded study design; patients are to be randomised into two parallel groups: receiving new community-based versus receiving hospital-based mental health care. Alongside the trials, a health economic evaluation will assess the cost-effectiveness of the intervention compared to care as usual, as well as assess the net benefits of the intervention across the 5 trials. To evaluate implementation outcomes and identify intervention and contextual factors that enhance sustainable implementation of community-based mental healthcare, a qualitative process evaluation will be carried out. Trial outcomes will be reported according to CONSORT recommendations.

### Site selection

Sites were selected based on the following criteria:

- the need for the and interest articulated by stakeholders to further develop and expand coverage of community care for people with mental ill health;
- concrete, explicit interest in the development and implementation of community-based mental health care by local, regional, and national stakeholders, including direct care providers, with interest demonstrated and documented in policy documents, political decisions, and/or through statements made on European Union platforms, such as the Joint Action for Mental Health and Wellbeing (2012–2015) [25];
- local leadership support for the implementation of service delivery changes in mental health care;
- a selection of sites that are at different stages of transition within the deinstitutionalisation process for mental health care; and
- human and technical resources available (as learned from site coordinators and through thorough situation analyses of each mental health system) to embark on the implementation of a community-based mental health team.

**Fig. 1:**
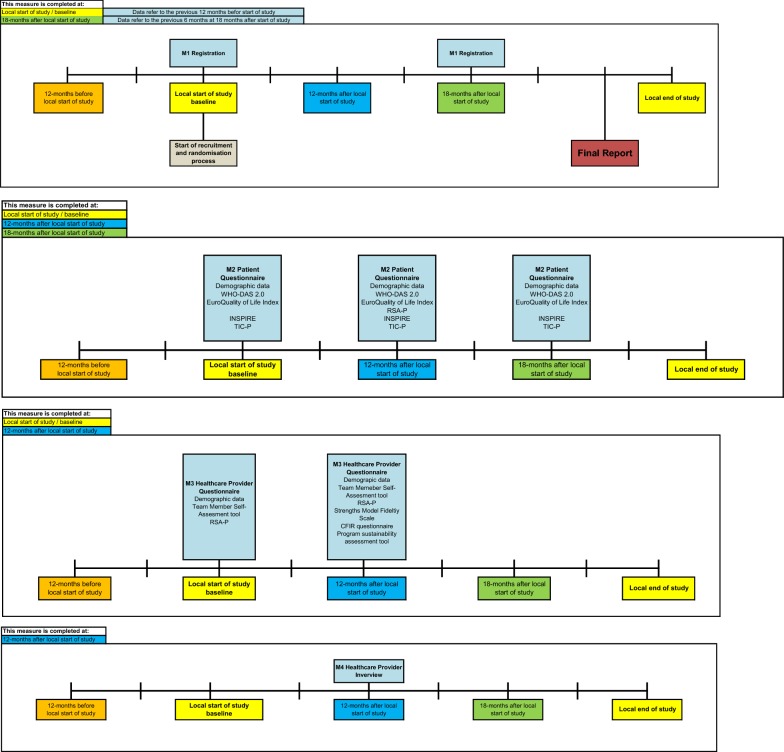
Timelines per RECOVER-E project site per outcome measure

Data will be collected in five cities in Central and Eastern Europe: Kotor, Montenegro; Skopje, North Macedonia; Sofia, Bulgaria; Siret-Suceava, Romania; and Zagreb, Croatia. Baseline, 12-month and 18-month follow-up data will be collected (Table 1). A summary of data collection points per measure is summarized in Fig. 1.

**Table 1:** RECOVER-E study timelines per implementation site

Site (city/county, country)	Local start of study	12 months after local start of study	18 months after local start of study
Sofia, Bulgaria	October 2019	October 2020	March 2021
Zagreb, Croatia	December 2018	December 2019	June 2020
Kotor, Montenegro	February 2019	February 2020	Aug 2020
Skopje, North Macedonia	June 2019	June 2020	January 2021
Suceava County, Romania	April 2019	April 2020	October 2020

### Study population

Participating service users are consenting adults (aged 18–65) with severe mental illness, defined as:

- meeting diagnostic criteria for bipolar disorder, severe major depression, schizophrenia, schizophreniform, and schizoaffective disorder according to the International Statistical Classification of Diseases and Related Health Problems (ICD-10), not in symptomatic remission and in need for continued care; and
- having severe limitations in personal and social role functioning (as per the International Classification of Functioning, Disability and Health, ICF), is not in functional remission and in need of coordinated care provided by community mental health teams.

The exclusion criteria for service users includes:

1. Service users under the age of 18;
2. Service users with dementia or other severe organic causes of brain damage that decreases their capacity to consent and participate in the study; and/or
3. Service users for whom treatment is legally prescribed (as is the case in forensic psychiatry).

In line with ethical requirements, any study participant can decide to withdraw from the intervention program, the research study, and/or one or more study measurements at any time. Study participants who have contributed data to the study will remain in the analysis (intention-to-treat principle). Data will be protected according to the General Data Protection Regulation [(EU) 2016/679)] and according to any additional local, regional, or national regulations that apply.

Participating health care providers include all members of the community mental health team at each clinic in the five countries, as well as service providers (i.e. psychiatrists, psychologists, nurses and social workers) providing care as usual to people with severe mental illness in each clinical setting in the five countries.

### Trial recruitment

The recruitment strategy employs sequential steps, with each subsequent recruitment step being applied when the previous step does not yield the targeted sample size. First, participants with an ICD-10 diagnosis of schizophrenia, schizophreniform, schizoaffective disorder, bipolar disorder, or severe major depression making a first entry into the mental health care system (i.e. first admissions without a prior treatment history) will be recruited in the study by a physician. If this recruitment strategy is fails to recruit the required sample size, the subsequent recruitment step will be to offer recruitment into the study to all service users who have a history of accessing mental health care and are making a re-entry into the mental health care system (i.e. re-admissions; patients that make a fresh start with a treatment for a new episode). If the targeted sample size cannot be not achieved using Strategies One and Two, service users in treatment for less than one year will become eligible for study inclusion and randomised to either the treatment or care as usual arm of the study.

All health care providers (e.g. physicians, nurses, and psychologists) involved in the teams will be recruited to participate in the study and to the intervention by the local research team at their site.

### Power and randomisation

With n = 90 per condition (intervention; usual care control), each of the five trials is powered to detect a clinically relevant effect (mean standardised difference) of d = 0.33 or greater (indicative of a medium sized effect) as statistically significant (at α ≤ 0.05, 2-tailed) with a power of (1 − β) = 0.80 when the primary outcome (WHODAS 2.0) is evaluated in a baseline adjusted analysis of variance (ANCOVA) or a similarly specified linear mixed model. Randomisation of consenting participants will be carried out by an independent statistician in each of the five sites, with patients as the unit of randomisation. Simple randomisation with 1:1 allocation will be applied using random.org for true random number generation. In this type of study (a hybrid implementation-effectiveness trial) it is not possible to conceal randomisation status neither from clinicians nor from patients, and masking will therefore not be attempted.

### Translation and cultural adaptation of measures

Language translation and the cultural adaption of measures will be conducted according to a five-step procedure. If translations do not currently exist, two independent translators fluent in English and the national language will translate the English-language versions into the local national language. The translated versions will be compared through a meeting of the two independent translators and a researcher from the implementation research team. Cultural adaptation is also considered in this meeting. The text in the national languages is translated back to English by an independent third translator. These versions will be compared to the original English-language versions in a meeting with all translators and a researcher from the implementation research team. The final version of the tool will be piloted with representatives from the target population. Changes will be made based on the pilot. Of note, several instruments have been validated in the implementation country or language prior to this project, and thus did not require cultural translation or adaptation.

### Intervention

The service delivery intervention entails the development and deployment of community-based service delivery alternatives for care provision in each of the five country sites through the development and implementation of multidisciplinary community-mental health teams (CMHT). Core members of the CMHT include at least one nurse, psychiatrist, psychologist, social worker and peer worker (person with lived experience of a severe mental illness). Adaptations to the CMHT staff structure may be required to reflect local needs and opportunities (e.g. one site may not have social workers but may have greater availability of nurses to work on the CMHT). This process of adaptation and tailoring will be by the clinical site coordinator, the local hospital management team, and relevant local care providers; advice and guidance on this process will be given by RECOVER-E implementation scientists. In order to avoid a top-down, directive approach, feedback and buy-in will be sought by the team at relevant points during the tailoring process. In order to increase scalability, transferability, and sustainability, the process of tailoring the intervention and implementation will be documented in an implementation log. As the trial is sequential, the data recorded in the log will serve to shape the teams and implementation in the later implementation sites.

The community mental health teams at each of the sites will be trained to deliver care within the flexible, assertive treatment (F-ACT) framework [26], enabling people with severe mental illness (SMI) to receive timely care in an appropriate intensity in the event of a crisis as well during stabilised periods. The F-ACT model’s workflows, staff requirements, and logistical considerations (technology, transport) are adapted to each site’s resources, with changes in the model documented across sites. In addition, the teams will be trained in adopting a strengths-based approach to care, transitioning to a therapeutic environment more aligned with principles of recovery-oriented care [27–32]. In line with the F-ACT model, in each country site, a CMHT team will work in a defined catchment area, serving all people within that catchment area with a diagnosis of severe depression, bipolar disorder, or schizophrenia. The training will serve not only to increase the skills of the CMHT, but will also give the teams an opportunity to discuss the F-ACT model and to tailor care pathways and methodologies for addressing the local situation and care needs.

Depending on individual patient needs, the CMHT can intensity or de-intensify care provision in two different ways:

- Individual case management by one member of the team, involving other providers from other disciplines depending on the needs of the patient.
- Flexible Assertive Community Treatment (F-ACT) team care forms a more intensive form of contact and care, where a patient has close contact with several members of the CMHT simultaneously (referred to as a shared caseload). These patients are listed on a digital Community Treatment Board [26], and are re-visited for care needs on a regular basis through CMHT team meetings. In team meetings, the CMHT discusses which form of care could be most helpful for the patient, review recovery goal attainment based on what the client has articulated, and distribute tasks for care provision (e.g. home visits, medication follow-up) among the team members. A patient is typically placed on the community treatment board if psychosis, severe depression, or bipolar disorder recurs, or threatens to recur, if hospitalisation is imminent, or if there is an emerging crisis or extenuating circumstances that may lead to a hospitalisation. Of note, a smaller proportion of patients fall under this category of more proactive care, compared to the proportion of the population served through individual case management.

The coordination and liaison work that CMHTs will do with primary care providers and other specialists (e.g. hospital-based specialised mental health care) will be defined locally through the development of care pathways in each implementation site, which will be developed with guidance from mental health and implementation experts on the RECOVER-E project team and with feedback from local stakeholders and care providers. Locally-tailored guidelines and protocols with options for evidence-based interventions (pharmacological, psychological, social) will be defined for each CMHT to use as well as for other providers outside of the CMHT to refer to. These locally-tailored guidelines and protocols will be documented, updated as innovations and changes in care occur at each site, and routinely shared between sites, as appropriate, in order to disseminate potential strategies and solutions, share knowledge, and increase the likelihood of sustainable, scaled-up community mental health interventions in Central and Eastern Europe.

A core set of evidence-based interventions for people with severe mental illness (SMI) can be delivered by community mental health teams, which include (but are not limited to) evidence-based interventions such as family-based motivational interviewing, individual motivational interviewing, and cognitive behavioural therapy.

### Usual care

Usual care consists of the existing mental health service provision model of that particular implementation site, typically involving inpatient mental health care as well as an institution-based outpatient mental health care. At the start of the RECOVER-E project, all site coordinators and clinicians in the project countries stated that none of their clinics in the five countries had specialised non-hospital based mental health professionals or formed CMHTs that structurally offer home treatment and/or crisis care in the community (Table 2).

**Table 2:** Usual care for severe mental illness in RECOVER-E project sites

Implementation site	Description of usual care for people with severe mental illness
1. Zagreb, Croatia	Specialised mental health care is provided primarily within hospitals (both in inpatient and outpatient settings). Pilot community mental health teams are being tested in several parts of Croatia, although not at this particular site. There is a community mental health centre in the city of Zagreb, which is broader in scope than the multidisciplinary community mental health team's work and focuses primarily on common mental disorders such as depression and anxiety. The primary interventions delivered as part of usual care include psychotherapy and medication. Treatment options available are dependent on the training and education of the provider.
2. Kotor, Montenegro	For people with severe mental illness, inpatient care is provided at this implementation site by the Psychiatric Hospital in Kotor, an outpatient care is provided by the mental health centre in Kotor (which is based in the primary care structure of the health system). The mental health centre accepts patients in its outpatient clinic and does not provide home-based treatment or crisis resolution outside of the clinic. The primary intervention that the mental heath centre provides is pharmacological intervention. Psychosocial rehabilitation is provided occasionally but not systematically. Inpatient admission is the standard procedure for any deterioration in mental health status and more than half of the beds in the psychiatric hospital are occupied by long-term care clients with severe mental illness from across the whole country.
3. Suceava County, Romania	Inpatient admission is the standard protocol during any deterioration in mental health status for people with SMI and there are currently no community mental health teams to provide home-based treatment or services in the community. The hospital has an occupational therapist but does not routinely work with people with SMI.
4. Skopje, North Macedonia	For people with severe mental illness, inpatient care is provided by the University Clinic of Psychiatry in Skopje (the nation's capital city), by the psychiatric hospital in Skopje or in a psychiatric ward in the city's general hospital. Specialised outpatient care is provided by a network of outpatient clinics. There are a few community mental health centres in North Macedonia. The community mental health centres accept patients in the office on an outpatient basis and do not provide home-based treatment or crisis resolution outside of the clinic. The primary intervention that the team provides is dispensing medication, supportive psychotherapy, psychosocial support and psychoeducation. Inpatient admission is the standard protocol during any deterioration in mental health status for clients with SMI.
5. Sofia, Bulgaria	People with SMI primarily receive medication from a psychiatrist in an outpatient department of the hospital. There are limited evidence-based psychosocial interventions provided to people with SMI and inpatient admission is the standard protocol during any deterioration in mental health status for clients with SMI.

#### Implementation strategies

The implementation strategies for implementing the CMHTs into practice have six components:

1. *Training*: Two weeks of training will be provided to CHMTs (1 week locally, 1 week as part of a hands-on study visit to the Netherlands). Health professionals will improve their understanding of community-based mental health approaches, with the goal of gaining the skills needed to become a cohesive CMHT in their city or district.
2. *Peer support*: Mental health providers who have received training to be a member of a CMHT will serve as an in-country resource for service delivery model principles and approaches. In addition, peer experts will constitute a novel introduction to the mental health workforce in the countries selected for implementation. This will strengthen the implementation of core values regarding service user inclusion and recovery among the team, and provide people with SMI experience-based (peer) support.
3. *Mentoring and supervision*: Skills learned have a greater likelihood of being applied and maintained over time when paired with ongoing coaching and consultation with mentors [33]. Based on training evaluations and ongoing implementation support calls, the clinical project lead in each site will develop mentoring and supervision plans with technical assistance from the international project team.
4. *Roadmaps:* Roadmaps will be a structured process for the CMHTs to define Specific, Mesaurable, Attainable, Relevant, Time-based (SMART), team-based goals on dimensions such as teamwork, treatment approaches, and recovery-oriented care. The roadmap includes milestones, timelines, and interventions to reach these goals. Roadmaps are followed-up every 6 months during a CMHT team meeting and through a progress review session with the coordinating institute of the project, where additional training and implementation support opportunities are agreed upon and organised.
5. *Audit and feedback:* Intervention and model fidelity are important components of a CMHT. Auditing the work and the skills/competences of the community mental health teams is important as a strategy for quality improvement, as well as for identify gaps that can be addressed in the future by the team.
6. *Pathway to scale:* To enhance sustainability of the CMHTs after the project period has ended, a pathway to scale for policy makers will be developed per site, obtaining information through two policy dialogues per country during the project period.

### Formative research to prepare for tailored implementation

A comprehensive assessment of current availability of mental health services (situation analysis) and needs assessment from stakeholders’ perspectives will be carried out in each implementation site. This is done through in-country site visits with the RECOVER-E implementation and mental health experts (expert panel) with the local teams.

Each site visit results in a plan of how local mental health professionals can use evidence-based interventions in ways that leverage local understandings and resources for the implementation of community-based mental health to be more sustainable and effective [34].

Data collection during site visits is ensured through extensive notes taken by each member of the expert delegation and implementation team during the site visits; a note taking template will be provided for each planned meeting and observation. The template will ensure that the reflections, thoughts, and insights of each member of the expert panel and implementation team are captured.

#### Consultation on current care systems and population

During site visits, the expert panel and implementation team will learn what specific aspects of care need to be transformed in order to allow for community mental health teams to function. This will result in a localised care pathway, to encourage and drive deliberate and explicit planning and collaboration around a clearly defined set of tasks, skills sets, and training strategies and accountability to enable, sustain, monitor and link their performance [34].

The situation analysis will include the following dimensions:

- Information about the catchment area
- Staff structure and caseload
- Roles and responsibilities of mental health professionals
- Triage processes
- Type of services and interventions available
- Financing of mental health services
- Existing opportunities to working with service users and persons with lived experience
- Collaboration of the health care institution with other stakeholders

These questions will be answered through a series of roundtable discussions and meetings with key stakeholders during the site visit, such as mental healthcare providers, other allied health care professionals, health care financiers (insurance companies, Ministries of Health), representatives of local councils or health authorities and service user and carer associations or networks. The findings from these roundtable discussions will be synthesised into a report and circulated for feedback through a consultative process with key stakeholders in that particular implementation site.

Further information will be collected by the local research lead through literature reviews, information gathered from the national statistical authority, findings from white papers and reports, and, when needed, from contact with stakeholders at the clinics and at the ministries of health.

#### Needs assessment

Once there is a clear understanding of the current care situation in each implementation site, an analysis of the current service delivery system in each clinic site will be carried out with the purpose of highlighting the determinants of implementation of the intervention, and understanding what needs service users and mental health providers have for receiving and delivering care. These needs and determinants provide subsequent inputs for the design of the training materials for community mental health teams. In particular, the following items will be assessed:

1. Unmet needs, desires and perceived preferences for care and recovery among service users (people with SMI) in each implementation site
2. Requirements (logistic, organizational, interpersonal) for being able to work as a provider within a multidisciplinary community mental health team

#### Implementation plan

Data collected provide the project team with a comprehensive understanding of local, contextual factors that may influence intervention implementation. Information from both the situation analysis and needs assessment will be used to develop a local implementation plan. The implementation plan will detail the context of the intervention (including local stakeholders, intervention target groups, cultural factors,) professional education and background of CMHT team members, and service user/patient characteristics; these questions will be adapted from the Tailored Implementation in Chronic Diseases (TICD) checklists [35]. The implementation plan is drafted by the local project teams, and include suggestions for tailoring the intervention to the local context. The process of tailoring the intervention will be documented; all adaptations will be recorded as well.

### Measures

#### Primary outcome

The primary outcome in the randomised trials is (personal and social) functioning, using the self-report 36-item version of the World Health Organization Disability Assessment Schedule 2.0 (WHODAS 2.0) [36]. The WHODAS 2.0 measures functional disability according to the Internal Classification of Functioning (ICF) framework. Importantly, functioning can be assessed across six life domains (cognition, mobility, self-care, getting along, life activities, and participation). The WHODAS 2.0 will be used as a continuous outcome in the clinical trial evaluation.

#### Secondary outcomes

The secondary patient-level outcome is health-related quality of life, measured with the 3-level EuroQoL (EQ-5D) [37, 38]. The EQ-5D-3L serves as the central outcome in the cost-utility analysis, which will be conducted as part of the health economic evaluation. The EQ-5D-3L is a widely used generic quality of life instrument, with the (older) 3-level version more commonly used in Central and Eastern Europe. The EQ-5D-3L contains 5 dimensions (mobility, self-care, daily activities, pain/discomfort and depression/anxiety) to describe a range of health states. Utility values can be calculated for these health states, using a valuation set elicited from the European population [37]. For sensitivity analyses, utility values will also be elicited using the EuroQoL’s visual analogue scale (VAS). The utility values give weight to the amount of time that a person spends in a certain health state, which helps to compute quality adjusted life years (QALYs), needed for the cost-utility analysis.

#### Resource use

Data on resource use (health care uptake), informal care, costs of travelling to health services, and productivity losses will be collected with a locally adapted version of the Trimbos/iMTA Questionnaire on Costs associated with Psychiatric illness, TiC-P [39]. Three types of cost categories are included: (1) costs stemming from health care uptake, (2) patients’ and their family’s (out-of-pocket) costs for co-payments, travel and informal care, (3) costs stemming from productivity losses due to absenteeism and lesser efficiency while at work. Costs will be estimated using a bottom-up (or micro-costing) approach, where units of health service are multiplied by their appropriate unit cost price and summed to provide an overall total cost estimate [40]. Cost will be measured in local currency, but for the economic evaluation converted to (international) euro using purchasing power parities (PPP) that at once consider exchange rates and the buying power in each of the countries. The reference year for the costs will be 2018.

#### Hospital (re)admission and length of stay

Data on the number of admissions to inpatient psychiatric care (per patient), and the duration (in days) of psychiatric inpatient admission will be collected at baseline (through analysis of the previous health record, if any) at 12-month and 18-month follow-up. Data will be extracted from hospital records and the records database of the CMHT by trained researchers external to the clinical site from the project team that have ethical approval to extract aggregated hospital data. Additional data at the service level (i.e. hospital or CMHT level) may include the number of patients receiving home treatment by the CMHT, number of total admissions per year in the hospital among people with SMI. This data will be collected at each research site by the local research lead from the records of the CMHTs and through aggregated hospital data.

#### Implementation outcomes

In addition to clinical and service use data, RECOVER-E will assess implementation outcomes. To guide the dimensions of implementation that the project will assess, the Reach, Effectiveness, Adoption, Implementation and Maintenance framework (RE-AIM) framework [41–43] will be used as a guiding implementation framework. RE-AIM enhances evaluation efforts and reduces the disparities between research, applied clinical practices, and sustainability of evidence-based practices over time. Implementation outcomes of interest are:

- *reach of the intervention among the target population in the 5 implementation sites*
  Using program data, the total number of people with severe mental illness (per site) that were approached to participate in the study will be collected, as well as the number of *people with SMI that accepted and declined to be randomised in the study to either the* intervention (CMHT) or usual care. In addition, the number of estimated people in the catchment area of the clinic with SMI will be analysed.
- *the adoption (uptake) of the intervention by service providers*
  To look at the extent to which the intervention is adopted by service providers (i.e. accepting their new roles and responsibilities, working as a multidisciplinary community team for the first time), the University of Washington AIMS Team Member Self-Assessment [44] tool will be administered to each member of the CMHT in the intervention arm of the study. The tool can be used to assess roles, current organisational capacity, comfort of executing tasks and future training needed for a variety of collaborative care tasks (identify and engage patients, initiate and provide treatment, track treatment outcomes, proactive care, other programmatic tasks) for mental health care. Qualitative data obtained through the process evaluation will complement the data obtained from the AIMS tool.
- *intervention fidelity*
  CMHTs will be trained to work in a recovery-oriented way and adopting a strengths-based approach in engagement with people with SMI. To assess the team’s alignment in care provision with the core tenets of a strengths-based approach, the Strengths Model Fidelity Scale [28, 45] will be administered at 12 month follow-up. The tool will be used to measure the implementation of the Strengths Model in case management programs. Each item on the scale is rated on the 5-point rating scale ranging from 1 (not implemented) to 5 (fully implemented). The scale is divided into 3 core areas: (1) structure, (2) supervision, and (3) clinical services. Fidelity items 1–2 relate to Structure; fidelity items 3–4 relate to Supervision; and fidelity items 5–9 relate to Clinical Services. High fidelity is achieved when a program reaches at least a 4 in all 3 areas.

The extent to which care provided by the CMHTs facilitates the recovery journey of people with SMI will assessed from the provider perspective using the Recovery Self-Assessment Scale (RSA-R) [7, 8] at 12-month follow-up. The RSA-R is a 36-item measure designed to gauge the degree to which programmes implement recovery-oriented practices. It is a self-reflective tool designed to identify strengths and target areas of improvement, as services strive to offer recovery-oriented care. The RSA has shown very good internal consistency in similar research environments (alpha = 0.96). Higher scores indicate that staff feel their workplace has a greater implementation of recovery-oriented practices [46, 47].

To explore the extent to which services received are recovery-oriented from the patient perspective, the INSPIRE tool [48] will be administered among service users to understand experiences of the support they receive from a mental health provider for their recovery. Service users will also be asked to assess the extent to which the services they receive and providers they engage with are recovery-oriented through the Recovery Self-Assessment (RAS-R, patient version) [49].

#### Sustainability

To gauge the extent to which decision-makers commit to continue the implementation of the intervention, the Program Sustainability Assessment tool (PSAT) [50] will be administered at 12-month follow-up by email, with a link to an online questionnaire to service providers, managers, and decision-makers in each site. The PSAT is a 40 item self-report tool with 8 sustainability domains that explore the level of sustainability of a public health intervention. To further support sustainability efforts, to bridge the gap between policy and practice, each country site will develop local policy influencing strategies. These strategies serve as a guide for implementation teams to frame the issues related to community mental health care that they want to engage policymakers on. Strategy development entails a brainstorming workshop to discuss policy issues related to mental health in the city/district/country, and stakeholder mapping to understand who to engage and how. On the basis of the strategy, an action plan with communication strategies for engaging policymakers on the policy ask (building a case for investing in CMHTs) will be developed. The messages articulated in the action plan will be applied in two policy dialogues, will be held in each implementation country. The aim of the policy dialogues is to bring stakeholders representing various interest groups to the same table. These policy dialogue sessions will serve as a vehicle through which stakeholders can see policy influencing problems from different perspectives, which in turn can help to improve a policy or plan. The second policy dialogue session will distil the findings from the research evaluation as further debate and create local policy impact, such as creating a case for investment into community-based alternatives for mental health support or the added value of having a peer expert as part of the mental health service delivery team. Policy dialogues will be run by the site coordinator in each country site in a face-to-face, roundtable meeting setting, using the contact information of stakeholders identified in the stakeholder mapping.

#### Process evaluation

To evaluate the implementation strategy chosen in our project, during implementation, we will carry out a process evaluation to capture the complexity inherent in mental health care services. This process evaluation will provide the project with a greater, more in-depth understanding of health systems in each implementation site and what is needed to improve them, as well as understanding why the intervention may be more or less effective in a certain implementation site [51]. The process evaluation will assess the implementation strategy regarding components such as organisational structure, political and cultural context, professional factors (including shift in roles and responsibilities, as well as dynamics between health care professionals, manager, clients, and carers). Clinicians and staff from the clinical sites, including members of the community mental health teams (CMHTs), will be asked to complete a questionnaire as well as participate in an interview, both based on the Consolidated Framework of Implementation Research (CFIR) interview guide [52]. Semi-structured interviews with a purposive sample of 5 providers from each site (from both the intervention and care as usual groups) will be conducted. The interviews will be recorded and analysed thematically, using a mix of inductive and deductive approaches, by a group of qualitative researchers in each project site.

#### Statistical data analysis

The five trials will be analysed separately as well as together on an intention-to-treat (ITT) basis, either using (generalised) mixed modelling or regression modelling on imputed data, with missing data multiply imputed using chained equations. In the primary analysis, the primary outcome (WHODAS 2.0 functioning) will be regressed on the treatment dummy (CMHT vs. CAU) with baseline WHODAS 2.0 as a covariate. Secondary outcomes will be analysed similarly. These analyses will answer the question to what extent the newly implemented community-based and recovery-oriented healthcare system has better patient-level outcomes with regard to WHODAS 2.0 functioning when compared to care as usual.

#### Health-economic evaluation

The health-economic evaluation (HEE) will be from the health service system perspective and apart from the societal perspective with costs for the reference year 2018. The HEE will conducted as a cost-effectiveness analysis with healthcare costs related to WHODAS 2.0 treatment response (recovered versus not recovered) and a cost-utility analysis of incremental costs per quality adjusted life years (QALY) gained. To simultaneously evaluate both costs and outcomes, seemingly unrelated regression equations (SURE) models will be used, and will be baseline-adjusted with baseline WHODAS 2.0, EQ-5D utilities and costs as covariates. Because costs are non-normally distributed the SURE models will be bootstrapped (2500 times). Incremental cost-effectiveness ratios (ICERs) will be computed. When most simulated ICERs fall into the NE quadrant of the ICER plane (indicating that better health is achieved at higher costs) then an acceptability curve will be graphed for decision-making purposes. The acceptability curve depicts the likelihood that one finds the new health care system more cost-effective than the former given varying willingness-to-pay (WTP) ceilings for gaining a QALY. One-way sensitivity analyses will be directed at uncertainties in cost drivers and outcomes. The HEE will be reported according to the CHEERS statement for trial-based health-economic evaluation.

### Ethics approval and data protection

A centralised study protocol was developed to coordinate the 5 independent trials. Each trial in each implementation site has a separate trial template, a locally adapted version of the centralised project study protocol, and a locally adapted version of the centralised project Data Management Plan. Ethical approval from institutional review boards will be obtained in each of the 5 trial countries prior to initiating any of the study procedures, including patient recruitment. All study protocols will comprise a dedicated section as to how to prevent the risk of enhancing vulnerability/stigmatization. Institutional review boards will be informed of any changes in protocol or methodology. An Independent Ethics Advisory Board with external experts to the project will meet regularly and have oversight over the overall ethical, safety, and data management and storage procedures of the trials.

## Discussion

The RECOVER-E project assesses the impact of implementing an evidence-based service delivery model for people with severe mental illness in different contexts in middle-income countries in Central and Eastern Europe. The project is focused on implementing evidence-based service delivery models in new sites that do not yet have a fully community-based recovery-oriented model of mental health services. It builds on the RECOVER-E Consortium’s experience in implementing this community-based model of care in other country contexts, where implementation of a new model of community-based mental health care has been challenging to implement and evaluate. It also builds on prior work of the Consortium in some of the RECOVER-E implementation sites (Croatia between 2016–2017 and Montenegro between 2013–2014), where it carried out policy work and capacity building activities preparing the ground for the RECOVER-E project roll out.

### Contributions to science

Evaluation of the implementation process as well as the effect of the intervention on patient-level outcomes will be done through 5 hybrid effectiveness-implementation trials with process evaluations and trial-based economic evaluations.

To increase ecological validity, the research team opted for pragmatic trials that help to evaluate the effectiveness of the intervention under real life conditions (as opposed to efficacy under well-controlled laboratory-like conditions). While this may have slightly affected internal validity (e.g. lack of blinding), the corresponding results may be more generalizable and applicable to routine practice settings. The pragmatic trials are also more suitable for economic evaluations, where one wants to probe the added value of the new intervention over and above routine practice.

In addition to effectiveness and health economic evaluation, an important goal of this project is to identify barriers and facilitators to implementation of community-based mental health services. The implementation process will be carefully monitored in real-time during the trial, with sequential structure with the start of the trials, and the possibility to make amendments to implementation strategies which can inform the start of the next site trial.

The RECOVER-E project will contribute to the growing body of research in mental health services development, particularly community-based mental health services for countries in transition or with limited availability of community-based mental health services.

### Contributions to society

Research findings and lessons learned from the implementation process will be transformed into a sustainability and scale-up plan for each site, to support the continuation of ongoing service delivery reforms towards community-based, recovery-oriented services. These lessons learned will form the basis for discussion in the policy dialogues planned in each of the 5 sites to help inform and engage policymakers about how such a community-based recovery-oriented model of services could inform further work at regional and national level to improve health and social outcomes for people with severe mental disorders. In addition, the RECOVER-E project introduces the function of a peer worker (i.e. person with lived experience of a severe mental disorder) in each of the community mental health teams across the five sites, which contributes to furthering the practice of peer workers as partners in mental health care provision.

### Strengths and limitations

There are several important strengths and limitations of this research project. Strengths include a robust evidence base and employing a rigorous evaluation design (randomised controlled trial) to assess patient outcomes, using validated measures. We also use a strong process evaluation and implementation research approach, which will aid in explaining why the intervention worked (or not) and to what extent it can be generalized to other settings. Finally, the RECOVER-E project benefits from a strong consortium of partners with local leadership and complementary expertise, needed to implement the project. There are several limitations that are important to mention. First, our study populations are heterogenous, looking across multiple diagnostic categories and country contexts, which means that care delivered by CMHT may work for some patients and not for others. Second, successful implementation is dependent on many contextual and culture factors including local legislation and policies for mental health care organization, motivation and competence of the team, hospital leadership where the CMHTs operate out of—many of these factors are not possible to change in the context of the project. It is also challenging to work across 5 languages and cultures (with other non-implementation project partners covering four other countries and languages), which makes qualitative research a particular challenge. To mitigate the impact of these challenges, the consortium discusses and works towards practical solutions for local implementation and research challenges through routine implementation and research calls. Finally, the project focuses on a single site, which impacts generalizability of findings when considering scale-up to additional sites in the country.

## Data Availability

RECOVER-E is a EU Horizon 2020 funded project participating in the Open Research Data Pilot. This pilot is part of the Horizon 2020 Open Access to Scientific Publications and Research Data programme. The goal of the program is to foster access to data generated in Horizon 2020 projects. As stated in the *ESiWACE Grant Agreement Article 29.3: Open Access to research data*, the RECOVER-E beneficiaries will ensure that data is open access; this will be done through open access peer- reviewed publications. Researchers from the RECOVER-E team will upload a copy of each peer- reviewed manuscript to an open access repository for publications before or within 6 months of publication; effort will be taken to upload manuscripts and data as soon as possible. Cleaned, anonymous, and analyzed data related to each publication will be uploaded to the repository, with cross-links to ensure ease of access.

## Abbreviations

CAU: Care as usual
CMHT: Community mental health team
F-ACT: Flexible assertive community treatment
HEE: Health economic evaluation
RCT: Randomised controlled trial
